# Organization and Implementation of a Stroke Center in Panamá, a Model for Implementation of Stroke Centers in Low and Middle Income Countries

**DOI:** 10.3389/fneur.2021.684775

**Published:** 2021-08-16

**Authors:** Nelson Novarro-Escudero, Yoon Ji Moon, Argelis Olmedo, Teresa Ferguson, Ileana Caballero, Eduardo Onodera, Euclides Effio, Lisa M. Klein, Elizabeth K. Zink, Brenda Johnson, Victor C. Urrutia

**Affiliations:** ^1^Primary Stroke Center, Pacífica Salud, Hospital Punta Pacífica, Panamá, Panama; ^2^Department of Neurology, Johns Hopkins University School of Medicine, Baltimore, MD, United States; ^3^Department of Nursing and Quality, Pacífica Salud, Hospital Punta Pacífica, Panamá, Panama; ^4^Department of Emergency Medicine, Pacífica Salud, Hospital Punta Pacífica, Panamá, Panama; ^5^Department of Radiology, Pacífica Salud, Hospital Punta Pacífica, Panamá, Panama; ^6^Department of Neurosurgery, Pacífica Salud, Hospital Punta Pacífica, Panamá, Panama; ^7^Department of Neurosciences, The Johns Hopkins Hospital Comprehensive Stroke Center, Baltimore, MD, United States

**Keywords:** stroke unit, acute stroke care, quality improvement, implementation, stroke centers

## Abstract

**Background:** Stroke is the second leading cause of death and disability worldwide. Stroke centers have become a central component of modern stroke services in many high-income countries, but their feasibility and efficacy in low, middle, and emerging high-income countries are less clear. Also, despite the availability of international guidelines, many hospitals worldwide do not have organized clinical stroke care. We present a methodology to help hospitals develop stroke centers and review quality data after implementation.

**Objectives:** To describe and compare demographics, performance, and clinical outcomes of the Pacífica Salud, Hospital Punta Pacífica (PSHPP) stroke center during its first 3 years 2017–2019.

**Methods:** Pacífica Salud, Hospital Punta Pacífica was organized to implement protocols of care based on the best practices by international guidelines and a quality improvement process. The methodology for implementation adapts a model for translating evidence into practice for implementation of evidence-based practices in medicine. This is a retrospective study of prospectively collected quality data between March of 2017 to December of 2019 for patients admitted to PSHPP with primary diagnosis stroke. Data collected include demographics, clinical data organized per the Joint Commission's STK Performance Measures, door to needle, door to groin puncture, 90 day modified Rankin Score, and hemorrhagic complications from IV thrombolysis and mechanical thrombectomy (MT). Primary outcome: year over year proficiency in documenting performance measures. Secondary outcome: year over year improvement.

**Results:** A total of 143 patients were admitted for acute ischemic stroke, TIA, or hemorrhagic stroke. Of these, 36 were admitted in 2017, 50 in 2018, and 57 in 2019. Performance measure proficiency increased in the year-over-year analysis as did the total number of patients and the number of patients treated with IV thrombolysis and MT.

**Conclusions:** We present the methodology and results of a stroke program implementation in Panamá. This program is the first in the country and in Central America to achieve Joint Commission International (JCI) certification as a Primary Stroke Center (PSC). We postulate that the dissemination of management guidelines is not sufficient to encourage the development of stroke centers. The application of a methodology for translation of evidence into practice with mentorship facilitated the success of this program.

## Introduction

Interventions of proven benefit for acute stroke treatment include: administration of intravenous thrombolysis (IVT) within 4.5 h of symptom onset (1–3), mechanical thrombectomy (MT) for large vessel occlusion (LVO) within 24 h for selected patients (4, 5), access to rehabilitation (6–8), aspirin, intensive secondary prevention (6, 9, 10), and stroke units (11–13). Acute interventions for ischemic stroke remain time dependent, with earlier treatment with IVT or MT being associated with lower disability, mortality, and complications (14, 15). In order to make these treatments available to all eligible patients, significant coordination, and streamlining of procedures for screening, evaluation, and treatment is needed.

Observational studies and randomized trials have shown that stroke units improve the use of diagnostic tests and acute treatments, increase access to rehabilitation services, and lengthen survival without dependence following acute ischemic stroke (11, 12, 16). Stroke units have become central components of modern stroke services in many high-income countries. Guidelines for organizing stroke units and performance measures to assess quality of stroke care have been developed based on randomized controlled trials, clinical practice guidelines, and expert consensus (3, 17–21).

While the guidelines are widely available, it is often difficult to implement stroke centers in regions such as emerging high-income countries and low- and middle-income countries (LMIC) where organized systems of care are uncommon, resources scarce, and experience limited. In this paper, we describe the development, implementation, and performance of a stroke program in a hospital in Panamá, which ultimately achieved Joint Commission International (JCI) certification as a Primary Stroke Center (PSC) in February of 2020.

## Methods

### Setting

This observational, retrospective analysis of prospectively acquired de-identified data from patients qualified (primary diagnosis stroke) for the stroke program at Pacífica Salud, Hospital Punta Pacífica (PSHPP) during the years 2017 through 2019. The hospital is a JCI accredited, private medical facility in Panamá City with 76 beds and over 5,500 inpatient visits per year.

### Methodology

In December of 2016, PSHPP and Johns Hopkins Medicine International started a collaboration to design and implement a stroke program. We followed a methodology that has been used by our group in other stroke program building projects, steps listed below. Our methodology is an adaptation of the process outlined by Pronovost et al. for translating evidence into practice (22, 23). This process was conducted with a minimum of monthly meetings over 6 months until the launching of the program. The process then continued with mentored touchpoints and mock surveys over the next 2.5 years until certification.

1. Guidelines and best practices to be implemented at the site were assessed.
2. Site survey was conducted to determine current stroke care, resources, staff, and logistics.
3. A multidisciplinary/interprofessional Stroke Task Force was formed to create policies, procedures, and workflows for the stroke program. The team consists of professionals from departments that provide care for stroke, including the Emergency Department, Radiology, Laboratory, Pharmacy, Intensive Care Unit, Inpatient Unit, Rehabilitation, Neurology, Neurosurgery, and Critical Care Medicine. Standardization of IV thrombolysis served as an anchor, setting the tone for interprofessional collaboration.
4. Intensive education program including lectures, training sessions, and reading material was implemented for nurses, physicians, healthcare workers, and all personnel in the hospital.
5. Apparatus for collecting data measuring the quality of the processes implemented was created.
6. Policies, procedures, and workflows were implemented and the stroke program was formally launched.
7. Collaborative efforts within the multidisciplinary/interprofessional task force continued. Progress and Quality Improvement (QI) process was monitored with the goal of achieving JCI certification. Mock surveys and data monitoring by the Stroke Task Force informed further QI.

### Data Collection

Data pertaining to the demographics, stroke performance measures, and stroke outcomes for adult patients (age ≥ 18) admitted to PSHPP with primary diagnosis stroke during the years 2017–2019 were collected prospectively, deidentified, and analyzed for quality improvement. Quality of stroke care was assessed using standard performance measures adapted from American Heart Association/American Stroke Association (24). We also assessed: stroke critical times, such as door to stroke protocol activation, door to neurological assessment, laboratory turnaround time, door to CT complete, door to CT read, door to needle, and door to groin (Table 1) (25–27); volume, complications of acute stroke interventions, and Thrombolysis In Cerebral Infarction scale (TICI) after thrombectomy; length of hospital stay and 90 day modified Rankin Score (mRS). Thrombolysis In Cerebral Infarction scores were determined restrospectively by EE, who reviewed the cerebral angiograms of patients undergoing MT. Times from groin puncture to recanalization were also determined retrospectively for 2019, which is when these data points were started to be documented.

**Table 1:** Stroke critical time goals.

**Time intervals**	**Goal Times (min)**
Door to physician evaluation^a^	10
Door to neurological evaluation by stroke team^b^	25
Door to lab	45
Door to CT completed	25
Door to CT read	20
Door to needle	60
Door to groin	90

a *Immediate general assessment by a stroke team, emergency physician, or other expert, including CT order*.

b *Emergency medicine trained physicians or neurologists were authorized to evaluate and administer IVT*.

### Statistical Analysis

Data are presented as proportions for dichotomous variables and mean [standard deviation] or median (interquartile range) for continuous variables. Significant *p*-values are *p* < 0.05.

Two-way ANOVA test was performed to compare means (i.e., mean age, baseline NIHSS) across years. Fisher exact test for proportions was performed to compare distribution of categorical variables (i.e., gender, stroke type) across years. For variables showing statistically significant *p*-value, an additional *post-hoc* analysis comparing each pair of years was conducted (Supplementary Table 2).

Fisher exact tests were performed for statistical analyses of stroke performance measures. For variables showing statistically significant *p*-value, an additional *post-hoc* analysis comparing each pair of years was conducted (Supplementary Table 3).

Two-way ANOVA tests were performed for statistical analyses of mean stroke critical times. Median quantile regression modeling was performed to obtain coefficient for median stroke critical times.

Fisher exact tests were performed for statistical analyses of percentage of complications from IV tPA or MT.

Two-way ANOVA tests were performed for statistical analyses of mean hospital stay.

All analyses were performed using STATA/IC 15.1.

### Ethical Considerations

This study was approved by the Johns Hopkins Institutional Review Board as well as the research Ethics Committee at PSHPP. Waiver of consent was granted given that it involved retrospective review of de-identified data collected for QI.

## Results

### Patient Demographics

**Figure 1:**
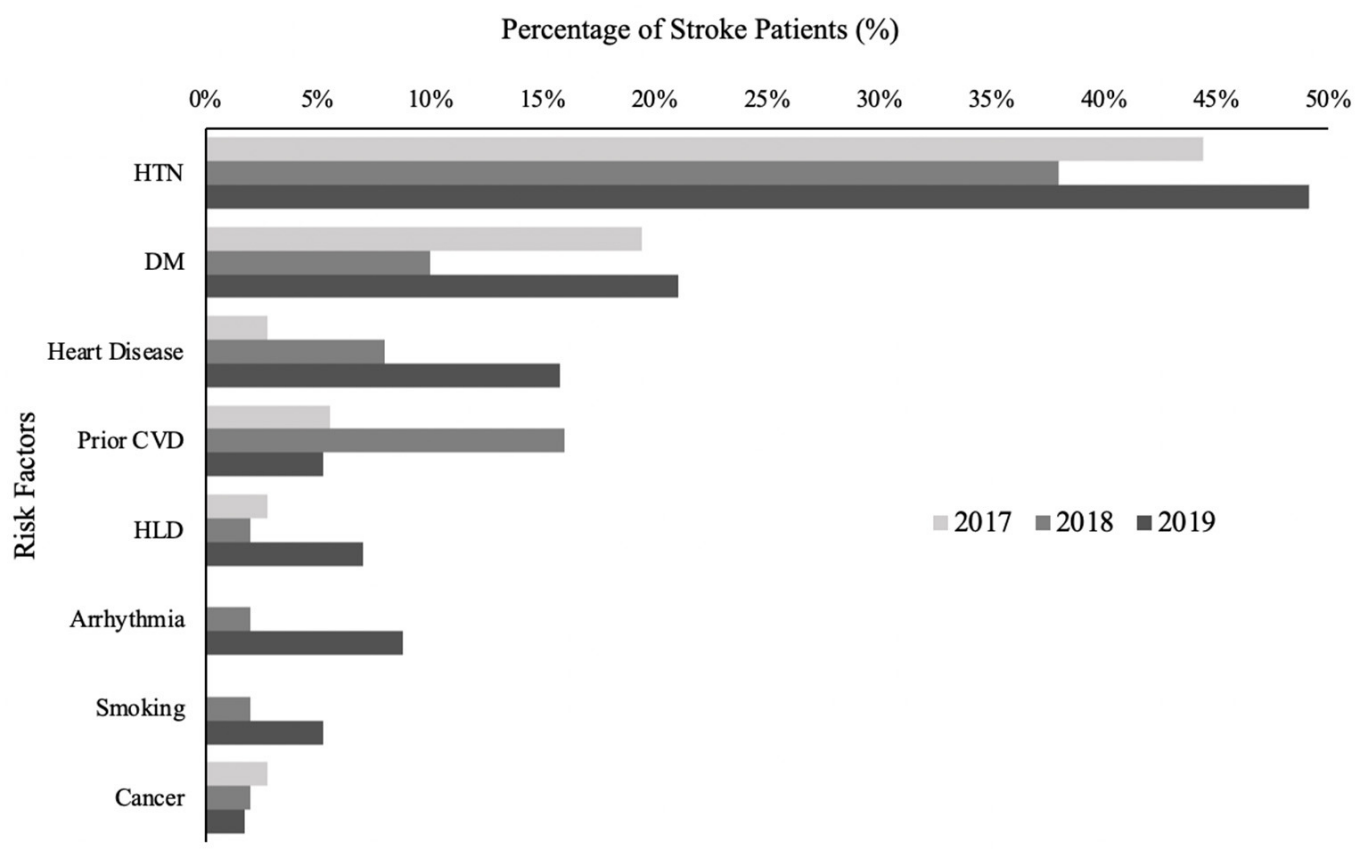
Stroke risk factors, %. Percentage of study population with given stroke risk factor. Prior CVD includes both stroke and TIA. Heart disease includes coronary artery disease, ischemic cardiomyopathy, valvular cardiomyopathy, dilated cardiomyopathy, and congenital cardiomyopathy. Arrhythmias include atrial fibrillation and supraventricular tachycardia.

A total of 143 patients were admitted for acute ischemic stroke, TIA, or hemorrhagic stroke; 36 were admitted in 2017, 50 in 2018, and 57 in 2019. There were no significant differences in sex (*p* = 0.112) among the 3 years (Table 2). Mean [SD] age of patients increased from 57.1 [16.4] in 2017 to 63.6 [17.6] in 2018 to 68.4 [16.7] in 2019. Distribution of stroke type was similar across years (*p* = 0.659), with TIA being the most common, hemorrhagic stroke the least common, and ischemic stroke consisting of about a third of all patients (Table 2). Mean [SD] baseline NIHSS for ischemic stroke patients was 8.5 [7.8] in 2017, 7.4 [5.0] in 2018, and 6.5 [5.1] in 2019 (*p* = 0.6725). Most common risk factors for stroke patients include hypertension, type II diabetes mellitus, heart disease, prior cerebrovascular disease, dyslipidemia, and arrhythmias (Figure 1).

**Table 2:** Patient demographics.

	**2017 *(n = 36)***	**2018 *(n = 50)***	**2019 *(n = 57)***	***p*-Value^**a**^**
**Gender**, ***n*****(%)**				
Male	15 (41.7%)	30 (60%)	36 (63.2%)	0.112
Female	21 (58.3%)	20 (40%)	21 (36.8%)	
**Age in years**, ***mean*****[SD]**	57.1 [16.4]	63.6 [17.6]	68.4 [16.7]	0.0087*^b^
**Stroke type**, ***n*****(%)**				
Ischemic	12 (33.3%)	20 (40%)	23 (40.4%)	0.659
TIA	16 (44.4%)	23 (46%)	28 (49.1%)	
Hemorrhagic	8 (22.2%)	7 (14%)	6 (10.5%)	
**Baseline NIHSS**, ***mean*****[SD]**				
Ischemic	8.5 [7.9]	7.4 [5.0]	6.5 [5.1]	0.6725
Hemorrhagic	6.8 [10.0]	2.5 [3.5]	17.7 [9.5]	0.1937

a Significant p-values are denoted with *(p < 0.05). Post-Hoc tests were performed using three independent, two-way t-tests with a Bonferroni adjusted alpha level of 0.016* (α/n = 0.05/3 = 0.016) when relevant.

b Post-Hoc analysis with Bonferroni adjusted alpha level of 0.016 reveal p-values of 0.0820 (2017 vs. 2018), 0.1584 (2018 vs. 2019), and 0.0019* (2017 vs. 2019).

### Performance Measures

**Figure 2:**
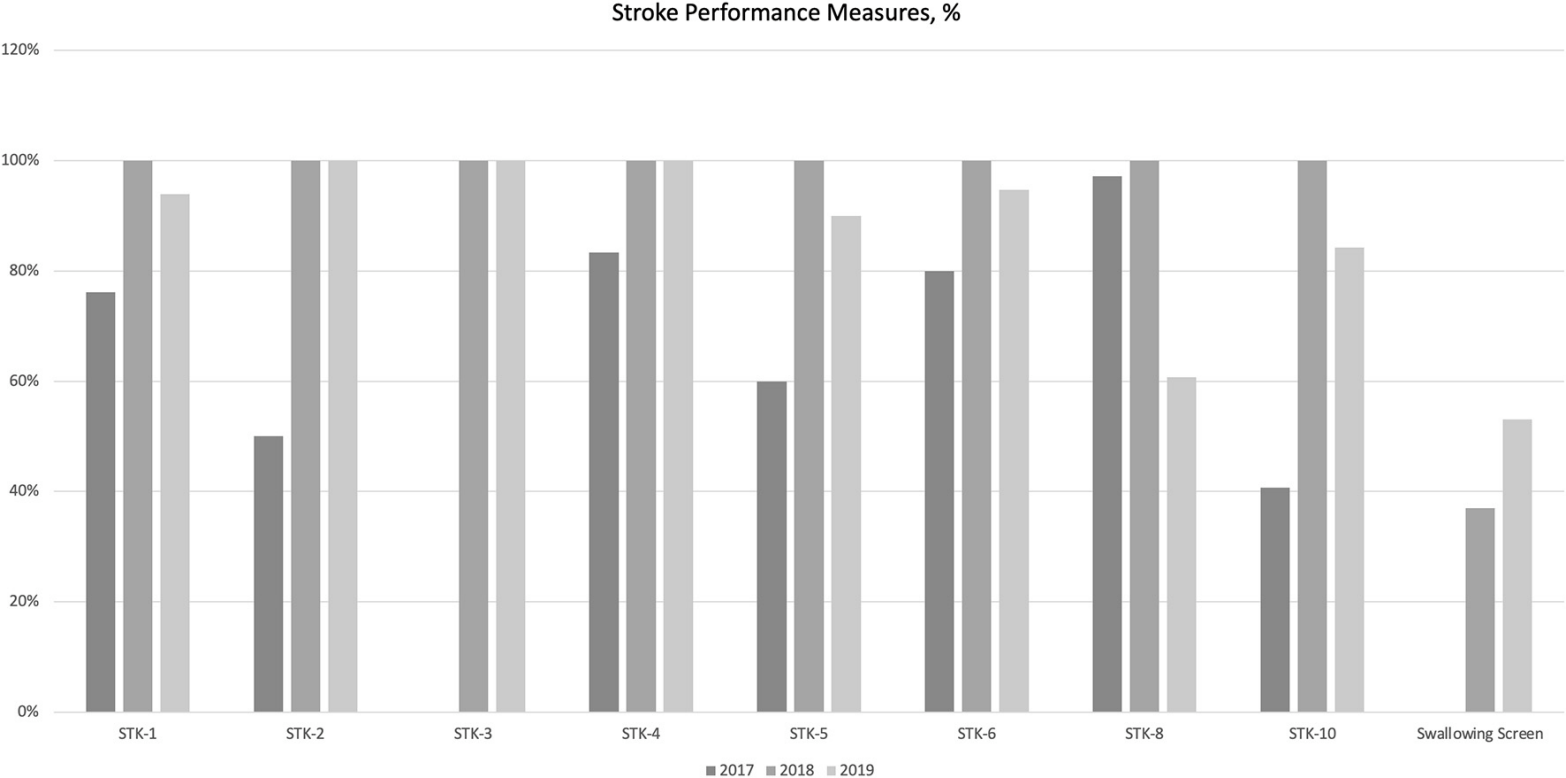
Stroke performance meassures 2017–2019.

In general, all stroke core measures (STKs) except stroke education (STK-8) showed an improvement between year 2017 and 2019. Similarly, the rate of swallowing screen steadily increased from 2017 to 2019, with the difference being significant for years 2017 vs. 2019 (Supplementary Table 3; Figure 2).

### Critical Time Measures

**Figure 3:**
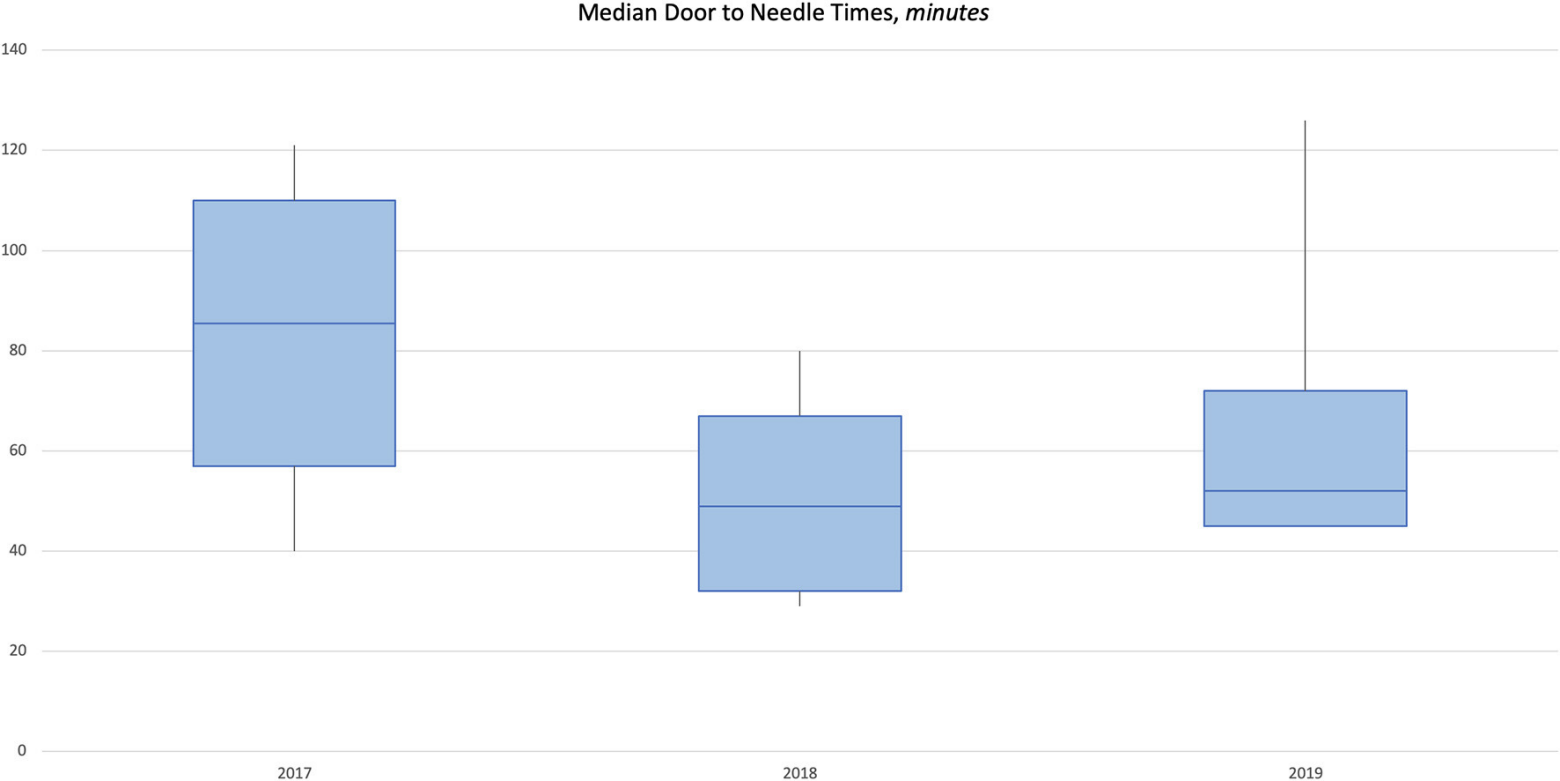
Median door to needle time 2017–2019.

**Figure 4:**
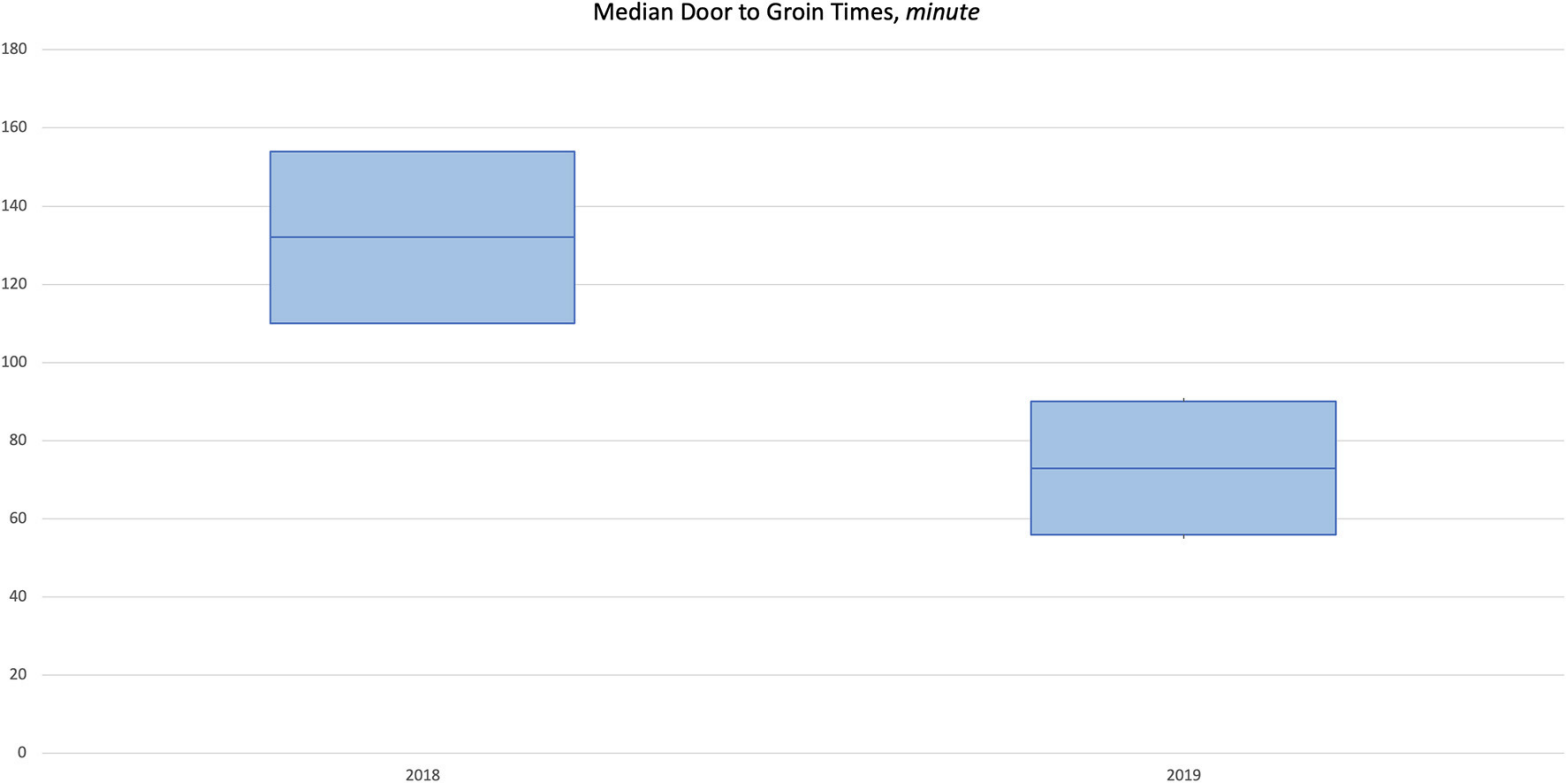
Median door to groin puncture 2017–2019.

There was a significant negative correlation between year and median door to neurological evaluation by neurologist/ED physician (coefficient = −12, *p* = 0.007) and a significant positive correlation between year and median lab turnaround time (coefficient = 8.5, *p* = 0.001; Supplementary Table 4). Door to needle time (min) median (IQR) was 85.5 (57–110) in 2017 and improved to 52 (45–72) in 2019 (Figure 3). Median (IQR) door to groin puncture (min) also improved from 132 (110–154) in 2018 to 73 (56–90) in 2019 (Figure 4). The time from groin puncture to recanalization was evaluated retrospectively. In 2018, these data points were not being documented and therefore not available. However in 2019, documentation of these time points became required and the median (range) for 2019 is 51 min(19–60). For all years, median door to stroke protocol activation time and median door to neurological assessment time met the goal of 10 and 25 min, respectively.

### Acute Stroke Intervention

The total number of patients receiving intravenous tPA steadily increased with year, from 6 in 2017 to 8 in 2018, and to 9 in 2019. There was one asymptomatic hemorrhage after IVT, a subdural hematoma, in 2017. The total number of patients receiving MT increased with year, with 0 in 2017, 2 in 2018, and 4 in 2019. The Thrombolysis In Cerebral Ischemia (TICI) scores were 2b or greater in all patients treated in 2018 and 2019. There was one case of fatal symptomatic intracerebral hemorrhage in 2019 (Table 3).

**Table 3:** Acute stroke interventions.

	**2017**	**2018**	**2019**	*p*-Value^*^
**Thrombolytic therapy (IV tPA)**				
Total number of patients receiving intravenous tPA, *n*	6	8	9	
Symptomatic intracerebral hemorrhage, *n* (%)	0	0	0	1.000
**Mechanical thrombectomy**				
Total number of patients receiving thrombectomy, *n*	0	2	4	
Symptomatic intracerebral hemorrhage, *n* (%)	n/a	0	1 (25%)	1.000
Thrombolysis in cerebral infarction (TICI), *mean* [SD]	n/a	3 [0]	2.8 [0.25]	0.3910

*Significant p-values are denoted with*

* *(p < 0.05)*.

### Outcome

**Figure 5:**
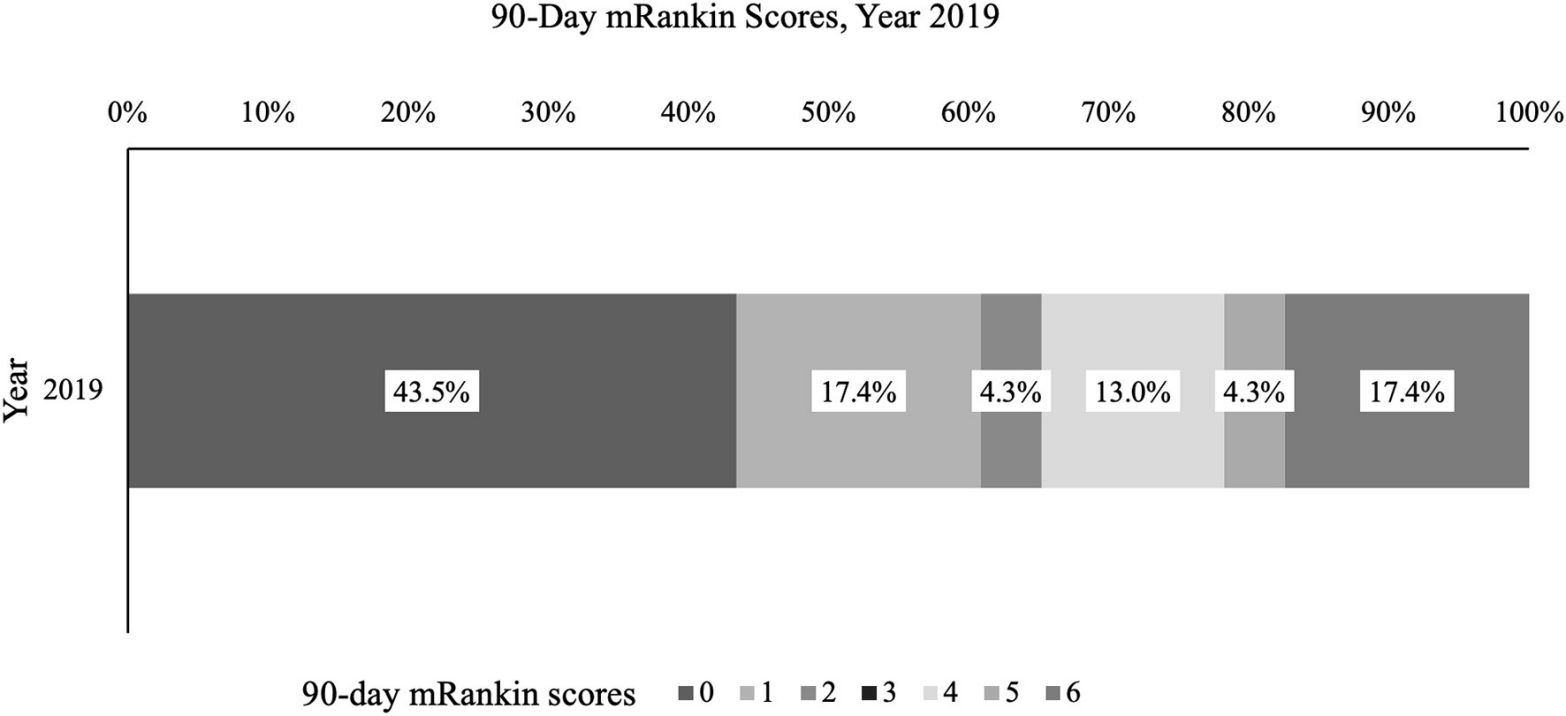
Ninety day modified Rankin Score, %. Percentage of stroke patients with given mRankin score for 2019 is presented.

There was no statistically significant difference in mean length of hospital stay across years, stratified by type of stroke (Supplementary Table 5). Mean (SD) length of hospital stay for any stroke patient was comparable year to year. Data for 90 day mRS was not available for all years as the collection of 90 day mRS was implemented in 2018; of those with documented mRS in 2019 (*n* = 23), 10/23 (43.5%) had a score of 0, 4/23 (17.4%) of 1, 1/23 (4.3%) of 2, 3/23 (13.0%) of 4, 1/23 (4.3%) of 5, and 4/23 (17.4%) of 6 (Figure 5).

## Discussion

We present the process for implementation of a stroke center in a hospital in Panamá. Over the development of the program, we observed a statistically significant improvement in a variety of standardized stroke performance measures: steady increase in the total number of stroke patients treated, and total number of IVT and MT in the year over year comparison. While data from before 2017 is not available as the QI database was created as part of this organizing effort; significant improvements are noted in most areas recorded during the first 3 years of implementation of the stroke center.

It is important to note, however, that not all trends reflect improvement in the data. Some unexpected findings may be the result of performance improvement projects which changed definitions of variables or measurement. For example, the stroke education procedure was redesigned in 2018, with a more rigorous definition of what qualified as “stroke education” and may explain the significant decrease in the rate of stroke education (STK-8) between the first 2 years and 2019. Also, the documentation of 90-day mRS was implemented in 2018; therefore, data for 2018 are sparse with significant improvements in documentation for 2019. The mRS in 2019 demonstrated a distribution consistent with published data (1, 28, 29). The turnaround time for laboratory results increased every year since 2017. The cause of this increase is not clear, however this underscores the importance of data collection and review as an integral process for stroke center development. Data needs to be monitored so that corrective measures can be instituted. A quality improvement project was started in 2020 to address this issue.

The door to needle time was significantly improved from 2017 to 2018 and maintained a median below 60 min. Door to groin puncture also improved from 2018 to 2019. The groin puncture to recanalization, median time, in 2019 was 51 min. All these critical times consistent with the literature and international benchmarks. There was only one symptomatic intracerebral hemorrhage among the thrombectomy patients, which was fatal. Due to the low number of cases, it is difficult to reach conclusions regarding the significance of this finding.

The organization of highly proficient stroke programs has been slower to spread in emerging high-income countries and LMIC, and in Latin America (30) despite widespread availability of guidelines such as the World Stroke Organization's Global Stroke Guidelines, Action Plan: A Roadmap for Quality Stroke Care, and materials made available by the Angel's initiative (31). One potential barrier is lack of experience, which in our project was overcome by an international collaboration functioning as mentors for PSHPP. It is also likely that implementation of guidelines in practice requires a methodology such as the one used by our group, which needs to be taught, again benefiting from a mentorship model for success. Teaching the methodology may be as important as having access to published guidelines.

A review of the literature concerning implementation of stroke units in LMIC or implementation of organized stroke care elements, did not reveal reports of a comprehensive methodological approach as was used in our group, rather; when discussing the development of stroke units, the emphasis is placed in the evidence for the benefit of stroke units, and the exortation that physicians need to take the lead to organize care teams and implement guidelines based on local resources (32). Papers on the topic tend to also concentrate on barriers and limitations in the applicability of guidelines in low resource environments, and not on a uniform methodology of successful implementation (33). Our work is novel in that it presents a comprehensive methodology to organize a PSC, which we believe will be a useful roadmap for implementation. One aspect of our experience that we believe is key, is the multidisciplinary and interprofessional approach which facilitates more comprehensive change, coupled with data collection to measure impact and pursue continuous quality improvement. Limited interventions may not yield the full benefits of stroke unit implementation (34).

Our implementation benefited from the setting. Panamá is an emerging high-income country and the setting is a small private hospital that has significant resources. This is in contrast with low resource settings in which barriers to stroke care are more significant. However, we view the value of the methodology in that it may be applied to different resource levels according to World Stroke Organization's Global Stroke Guidelines, Action Plan: A Roadmap for Quality Stroke Care (31), after determining the resource level, the implementation would follow the methodology described in this paper. This will need to be ultimately tested in different resource levels.

This study is limited due to the program being implemented in a small private hospital in one country. The findings in this study, therefore, should not be used to draw conclusions about the general patient population in Latin America. Other limitations include the small total volumes and the lack of pre implementation data which make it difficult to ascertain the effect of organization over the baseline stroke care proficiency at this hospital. Finally, challenges in data definition and collection are also noted.

In conclusion, we present a methodology and results of the implementation of a stroke program in Panamá. This program is the first in the country and in Central America to achieve JCI certification as a PSC. We postulate that the dissemination of management guidelines is not sufficient for the development of stroke centers, but the application of a methodology for translation of evidence into practice with mentorship can facilitate success of the program.

## Data Availability Statement

The raw data supporting the conclusions of this article will be made available by the authors, without undue reservation.

## Ethics Statement

The studies involving human participants were reviewed and approved by Johns Hopkins University School of Medicine and Pacífica Salud Hospital Punta Pacífica. Written informed consent for participation was not required for this study in accordance with the national legislation and the institutional requirements.

## Author Contributions

NN-E: led implementation, manuscript review, and analysis. YM: manuscript first draft, statistics, and analysis. AO: implementation, data collection, and manuscript review. TF, IC, EO, EE, LK, EZ, and BJ: implementation and manuscript review. VU: designed implementation methodology, led implementation, manuscript review, writing segments of manuscript, and analysis. All authors contributed to the article and approved the submitted version.

## Conflict of Interest

VU—Site PI for TIMELESS (Genentech Inc), Grant for investigator sponsored trial OPTIMISTmain (Genentech, Inc.). The remaining authors declare that the research was conducted in the absence of any commercial or financial relationships that could be construed as a potential conflict of interest.

## Publisher's Note

All claims expressed in this article are solely those of the authors and do not necessarily represent those of their affiliated organizations, or those of the publisher, the editors and the reviewers. Any product that may be evaluated in this article, or claim that may be made by its manufacturer, is not guaranteed or endorsed by the publisher.
