# Supplementary material for: Organization and Implementation of a Stroke Center in Panamá, a Model for Implementation of Stroke Centers in Low and Middle Income Countries
Source: Front Neurol. 2021 Aug 16;12:684775. doi: 10.3389/fneur.2021.684775 (PMC8415350; doi:10.3389/fneur.2021.684775)
Supplement: Supplementary file 1 [file Table_1.PDF]

Supplementary Material

1 Supplementary Tables

Table S1. Stroke Performance Measures, adapted from AHA/Brain Attack Coalition.

**STK-1. VTE prophylaxis[2]**	
**Definition**	Percentage of ischemic or hemorrhagic stroke patients prescribed VTE prophylaxis on hospital day 0 or 1
**Numerator**	Ischemic or hemorrhagic stroke patients prescribed VTE prophylaxis on the day of or day after admission
**Denominator**	**Included:** All patients with ischemic or hemorrhagic stroke<br>**Excluded:** < 18 years old, length of stay < 2 days, length of stay > 120 days, "comfort measures only," enrolled in clinical trials related to stroke, admitted for "elective carotid intervention"
**STK-2: Discharged on antithrombotic therapy**	
**Definition**	Percentage of patients with ischemic stroke who are discharged on antithrombotic
**Numerator**	Ischemic stroke patients prescribed antithrombotic therapy at hospital discharge
**Denominator**	**Included**: All patients with ischemic stroke<br>**Excluded**: < 18 years old, length of stay > 120 days, "comfort measures only," enrolled in clinical trials related to stroke, admitted for "elective carotid intervention," discharged to another hospital, left AMA, expired, discharged to home or health care facility for hospice care, documented reason for not prescribing antithrombotic at discharge
**STK-3: Anticoagulation therapy for atrial fibrillation/flutter**	
**Definition**	Percentage of patients with ischemic stroke and AF/atrial flutter who are discharged on anticoagulation therapy
**Numerator**	Ischemic stroke patients prescribed anticoagulation therapy at hospital discharge
**Denominator**	**Included**: Ischemic stroke patients with documented AF or atrial flutter<br>**Excluded**: < 18 years old, length of stay > 120 days, "comfort measures only," enrolled in clinical trials related to stroke, admitted for "elective carotid intervention," discharged to another hospital, left AMA, expired, discharged to home or health care facility for hospice care, documented reason for not prescribing anticoagulation at discharge
**STK-4: Thrombolytic therapy**	
**Definition**	Percentage of patients with acute ischemic stroke who arrived at hospital within 2 hours of time last known well for whom intravenous tPA was initiated at this hospital within 3 hours of time last known well
**Numerator**	Ischemic stroke patients for whom an intravenous thrombolytic therapy regimen was initiated at this hospital within 3 hours of time last known well
**Denominator**	**Included**: Acute ischemic stroke patients whose time of arrival is within 2 hours of time last known well<br>**Excluded**: < 18 years old, length of stay > 120 days, "comfort measures only," enrolled in clinical trials related to stroke, admitted for "elective carotid intervention," time last known well to ED arrival > 2 hours, documented reason for extending initiation of or not initiating IV thrombolytic
**STK-5: Antithrombotic therapy by end of hospital day 2**	
**Definition**	Percentage of patients with ischemic stroke who had antithrombotic therapy by end of hospital day 2
**Numerator**	Ischemic stroke patients who had antithrombotic therapy by end of hospital day 2
**Denominator**	**Included**: All patients with ischemic stroke

	**Excluded**: < 18 years old, length of stay < 2 days, length of stay > 120 day, "comfort measures only," enrolled in clinical trials related to stroke, admitted for "elective carotid intervention," time last known well to ED arrival > 2 hours, documented reason for extending initiation of IV thrombolytic, received IV or IA thrombolytic therapy at this hospital or within 24 hrs prior to arrival, documented reason for not prescribing antithrombotic by end of hospital day 2
**STK-6: Discharged on statin medication**	
**Definition**	Percentage of patients with ischemic stroke who are discharged on statin medication
**Numerator**	Ischemic stroke patients prescribed statin at hospital discharge
**Denominator**	**Included**: Ischemic stroke patients with a low-density lipoprotein cholesterol (LDL-C) ≥100 mg/dL OR LDL-C not measured OR who were taking a lipid-lowering medication before hospital arrival<br>**Excluded**: < 18 years old, length of stay > 120 days, "comfort measures only," enrolled in clinical trials related to stroke, admitted for "elective carotid intervention," left AMA, expired, discharged to home or health care facility for hospice care, documented reason for not prescribing statin at discharge
**STK-8: Stroke education[2]**	
**Definition**	Percentage of ischemic or hemorrhagic stroke patients who receive stroke education before hospital discharge
**Numerator**	Ischemic or hemorrhagic stroke patients or caregivers who receive educational materials addressing:<br>1. Activation of emergency medical system<br>2. Follow-up after discharge<br>3. Medications prescribed at discharge<br>4. Risk factors for stroke<br>5. Warning signs and symptoms of stroke
**Denominator**	**Included:** All patients with ischemic stroke<br>**Excluded:** < 18 years old, length of stay > 120 days, "comfort measures only," enrolled in clinical trials related to stroke, admitted for "elective carotid intervention", discharge to another acute care hospital, left AMA, expired, discharged to home or health care facility for hospice care, documented reason for not providing stroke education at discharge
**STK-10: Assessed for rehabilitation[2]**	
**Definition**	Percentage of ischemic or hemorrhagic stroke patients assessed for, or who received, rehabilitation services
**Numerator**	Ischemic or hemorrhagic stroke patients assessed for, or who received, rehabilitation services during the hospital stay
**Denominator**	**Included**: All patients with ischemic stroke<br>**Excluded**: < 18 years old, length of stay > 120 days, "comfort measures only," enrolled in clinical trials related to stroke, admitted for "elective carotid intervention," discharged to another hospital, left AMA, expired, discharged to home or health care facility for hospice care
**Swallowing Screen**	
**Definition**	Percentage of stroke patients for whom dysphagia screening was performed within 24 hours of admission using a dysphagia screening tool approved by institution
**Numerator**	Ischemic or hemorrhagic stroke patients for whom dysphagia screening was performed
**Denominator**	**Included:** All patients with ischemic or hemorrhagic stroke<br>**Excluded:** < 18 years old, length of stay > 120 days, stroke occurred while in hospital, enrolled in clinical trials related to stroke, admitted for "elective carotid intervention," discharged before 24 hours, documented reason that screening was not indicated

2 Post-Hoc Tests via Bonferroni Correction

Table S2 Post-Hoc Analysis for Demographics. For the demographics variable that showed statistically significant p-value for initial analysis (Table 2), post-hoc analyses using fisher exact tests for categorial variables and two-way, unpaired T-tests for continuous variables were performed to

compare each pair of years (2017 vs. 2018, 2018 vs. 2019, 2017 vs. 2019). A Bonferroni adjusted alpha level of 0.016 (α/n = .05/3 = 0.016) was used and significant p-values with adjusted alpha level are denoted with *.

	p-values		
	2017 vs 2018	2018 vs. 2019	2017 vs. 2019
**Mean age**	0.0820	0.1584	0.0019*

Table S2 Post-Hoc Analysis for Stroke Performance Measures. For the stroke performance measures that showed statistically significant p-value for initial analysis (Table S3), post-hoc analyses using fisher exact tests for categorical variables and two-way, unpaired T-tests for continuous variables were performed to compare each pair of years (2017 vs. 2018, 2018 vs. 2019, 2017 vs. 2019). A Bonferroni adjusted alpha level of 0.016 (α/n = .05/3 = 0.016) was used and significant p-values with adjusted alpha level are denoted with *.

	p-values		
	2017 vs 2018	2018 vs. 2019	2017 vs. 2019
**STK-1**	0.006*	0.239	0.071
**STK-2**	0.006*	--	0.002*
**STK-3**	0.125	--	0.003*
**STK-5**	0.024	0.360	0.076
**STK-8**	0.427	<0.001*	<0.001*
**STK-10**	<0.001*	0.115	0.003*
**Swallowing screen**	<0.001*	0.136	<0.001*

Table S3. Performance Measures, (%)

Performance Measures, %	2017	2018	2019	*p*-value[1]
STK-1: VTE Prophylaxis [2]	16/21 (76.2)	34/34 (100)	31/33 (93.9)	0.004*
STK-2: Discharged on Antithrombotic [3]	5/10 (50)	14/14 (100)	20/20 (100)	< 0.001*
STK-3: Anticoagulation for Afib/flutter [3]	0/7 (0)	1/1 (100)	4/4 (100)	0.001*
STK-4: Thrombolytic Therapy [3]	5/6 (83.3)	3/3 (100)	4/4 (100)	1.000
STK-5: Antithrombotic by Hospital Day 2 [3]	6/10 (60)	13/13 (100)	18/20 (90)	0.035*
STK-6: Discharged on Statin [3]	8/10 (80)	12/12 (100)	18/19 (94.7)	0.225
STK-8: Stroke Education [2]	34/35 (97.1)	47/47 (100)	34/56 (60.7)	< 0.001*
STK-10: Assessed for rehabilitation [2]	11/27 (40.7)	19/19 (100)	16/19 (84.2)	< 0.001*
Swallowing Screen[2]	0/35 (0)	10/27 (37.0)	26/49 (53.1)	< 0.001*

[1] Significant p-values are denoted with * (p < 0.05). Post-hoc tests were performed when relevant using the Bonferroni adjusted alpha level of 0.016.

[2] Included all stroke patients (ischemic, TIA, hemorrhagic) for calculation of these performance measures.

[3] Included ischemic patients for calculation of these performance measures.

Table S4. Stroke Critical Times, median (Interquartile range), *minutes*

**Median Time (IQR),** ***minutes***	**2017** *(n = 36)*	**2018** *(n = 50)*	**2019** *(n = 57)*	**Coefficient**[3]	***p*-value**[3]
Door to Stroke Protocol Activation[1]	1 (0 - 5)	10 (0 – 21)	1 (0 – 8)	-2.2e-16	1.000
Door to Neurological Evaluation by Neurologist/ED Physician[2]	20 (20 - 20)	20 (3 – 43)	3 (0 - 11)	-12	0.007*
Lab Turnaround Time	42 (35 – 53)	52 (45 – 65)	58 (43 - 68)	8.5	0.001*
Door to CT order	15 (6 - 30)	19.5 (9 - 43)	14 (7 - 24)	-0.5	0.837
Door to CT complete	36 (21 - 45)	31 (19 - 56)	34 (22 - 52)	-1	0.781
Door to CT read	38 (26 – 65)	47 (35 – 71)	48 (34 – 80)	5	0.286
Door to needle	85.5 (57 - 110)	49 (32 - 67)	52 (45- 72)	-13.5	0.440
Door to groin	None	132 (110 - 154)	73 (56 - 90)		

[1] Immediate general assessment by stroke team, ED physician, or other expert. Goal is within 10 minutes of arrival.

[2] Neurological assessment by stroke team, ED physician, or other expert. Goal is within 25 minutes of arrival.

[3] Median quantile regression modeling was performed. Significant p-values are denoted with * ($p < 0.05$).

Table S5. Length of Stay, *days*

	**2017**	**2018**	**2019**	***p*-value**[1]
**Hospital stay,** *mean days* [SD]				
Ischemic	3.8 [2.7]	5.6 [7.5]	6.9 [13.2]	0.6974
TIA (< 24 hours)	1.3 [1.3]	2.1 [1.6]	1.8 [1.9]	0.3556
Hemorrhagic	10 [10.8]	16.7 [30.2]	7.5 [11.2]	0.6784
Any stroke	4.1 [6.2]	5.5 [12.6]	4.4 [9.4]	0.7759

[1] Significant p-values are denoted with * ($p < 0.05$).
